# MicroRNA-329-3p inhibits the Wnt/β-catenin pathway and proliferation of osteosarcoma cells by targeting transcription factor 7-like 1

**DOI:** 10.32604/or.2023.044085

**Published:** 2024-02-06

**Authors:** HUI SUN, MASANORI KAWANO, TATSUYA IWASAKI, ICHIRO ITONAGA, YUTA KUBOTA, HIROSHI TSUMURA, KAZUHIRO TANAKA

**Affiliations:** Department of Orthopaedic Surgery, Faculty of Medicine, Oita University, Oita, 879-5503, Japan

**Keywords:** MiR-329-3p, TCF7L1, Wnt/β-catenin pathway, Osteosarcoma, Proliferation

## Abstract

An important factor in the emergence and progression of osteosarcoma (OS) is the dysregulated expression of microRNAs (miRNAs). Transcription factor 7-like 1 (TCF7L1), a member of the T cell factor/lymphoid enhancer factor (TCF/LEF) transcription factor family, interacts with the Wnt signaling pathway regulator β-catenin and acts as a DNA-specific binding protein. This study sought to elucidate the impact of the interaction between miR-329-3p and TCF7L1 on the growth and apoptosis of OS and analyze the regulatory expression relationship between miRNA and mRNA in osteosarcoma cells using a variety of approaches. MiR329-3p was significantly downregulated, while TCF7L1 was considerably up-regulated in all examined OS cell lines. Additionally, a clinical comparison study was performed using the TCGA database. Subsequently, the regulatory relationship between miR-329-3p and TCF7L1 on the proliferation and apoptosis of OS cells was verified through *in vitro* and *in vivo* experiments. When miR-329-3p was transfected into the OS cell line, the expression of TCF7L1 decreased, the proliferation of OS cells was inhibited, the cytoskeleton disintegrated, and the nucleus condensed to form apoptotic bodies. The expression of proteins that indicate apoptosis increased simultaneously. The cell cycle was arrested in the G0/G1 phase, and the G1/S transition was blocked. The introduction of miR-329-3p also inhibited downstream Cyclin D1 of the Wnt pathway. Xenograft experiments indicated that the overexpression of miR-329-3p significantly inhibited the growth of OS xenografts in nude mice, and the expression of TCF7L1 and c-Myc in tumor tissues decreased. MiR-329-3p was significantly reduced in OS cells and played a suppressive role in tumorigenesis and proliferation by targeting TCF7L1 both *in vitro* and *in vivo*. Osteosarcoma cell cycle arrest and pathway inhibition were observed upon the regulation of TCF7L1 by miR-329-3p. Summarizing these results, it can be inferred that miR-329-3p exerts anticancer effects in osteosarcoma by inhibiting TCF7L1.

## Introduction

Osteosarcoma (OS), characterized by extreme invasiveness and high mortality, is predominant primary bone malignancy; it has a distinctive bimodal age distribution, with its initial peak occurring at 10–20 years and a second peak at >60 years [1]. While significant advancements have been achieved in treating localized OS, with 60%–70% of patients attaining a 5-year survival, the prognosis for individuals with metastatic or recurrent disease remains disheartening, with survival rates plummeting to 20%–30% and 20%, respectively [2,3]. The urgent need to identify and explore novel targets for OS treatment is of paramount importance, as it holds the potential to significantly improve outcomes and enhance the quality of life for individuals affected by this devastating ailment.

MicroRNAs (miRNAs) are short, single-stranded RNA molecules, typically 21–25 nucleotides in length [4]. Despite their non-coding nature, these endogenous molecules play crucial roles in various biological processes [5,6]. MiRNAs are aberrantly expressed in different tumors, indicating their indispensable roles in tumorigenesis and tumor progression [7]. Among these, microRNA-329-3p (miR-329-3p) emerges as a tumor suppressor located at 14q32.31 on the human chromosome [8]. Its expression is downregulated in glioblastoma, hepatocellular carcinoma, colorectal cancer, and cervical cancer [9–12]. Additionally, miR-329-3p is reported to regulate tumor cell invasion and differentiation through interaction with target genes [13].

Transcription factor 7-like 1 (TCF7L1, often referred to as TCF3) belongs to the T cell factor/lymphoid enhancer factor (TCF/LEF) family. TCF7L1 acts as a DNA-specific binding protein, inducing the transcription of Wnt target genes by interacting with the regulator of the Wnt signaling pathway, β-catenin [14,15]. Various classes of tumor tissues exhibit upregulated expression of TCF7L1, which modulates tumor biological behavior and may be associated with poor prognosis. For instance, in prostate cancer, elevated TCF7L1 expression leads to IL-8/CXCR2 signaling-driven neuroendocrine differentiation and cell migration, promoting malignancy [16]. In colorectal cancer, TCF7L1 positively regulates cell proliferation, and suppressing its expression in xenografts results in tumor size reduction [17]. BCR-ABL-positive acute lymphoblastic leukemia with high TCF7L1 expression is associated with poor prognosis, and TCF7L1 knockdown significantly reduces cell proliferation and colony formation [18]. Silencing TCF7L1 suppresses the viability of gastric cancer cells, while overexpressing it promotes cell proliferation [19]. Although these studies primarily focused on the effects of TCF7L1 on tumor cell proliferation and identified several miRNAs targeting TCF7L1, there is a pressing need for more in-depth investigation into the regulation of TCF7L1 by miRNA in OS.

Compared to human mesenchymal stem cells (hMSCs), osteosarcoma cells express significantly less miR-329-3p and more TCF7L1. However, whether there is a true negative regulation between the two has not been experimentally verified. This study will focus on the regulation between the two and their roles in the Wnt/β-catenin pathway. By potentially interacting with TCF7L1 and the Wnt pathway, miR-329-3p may represent a candidate for modulating the growth of OS cells.

## Materials and Methods

### Cell lines

The RIKEN Cell Bank in Tsukuba, Japan, provided the human OS cell lines MG-63, SaOS-2, and HOS, while the JCRB Cell Bank in Osaka, Japan, provided NY and Hu09. Human mesenchymal stem cells (hMSCs) were acquired from Takara Bio Inc. (Shiga, Otsu, Japan) with appropriate ethical approval. Each cell line’s genotype and phenotype were verified by the firm that provided it. MG-63, SaOS-2, and NY cells were cultured in DMEM High Glucose (Gibco, Thermo Fisher K.K., Tokyo, Japan), while HOS cells were grown in MEM (Gibco) supplemented with 10% FBS (Gibco). The Hu09 cells were cultured in RPMI-1640 (Gibco) with 10% FBS. Human MSCs were cultivated using MSCGM-CD Medium (Takara Biotechnology, Shiga, Japan). All cells were passaged every two to three days and kept at 37°C with 5% CO_2_.

### MiRNA and mRNA expression analysis using microarrays

In this study, miRNA and total RNA were extracted from triplicate samples of five distinct OS cell lines (MG-63, SaOS-2, HOS, NY, and Hu09) and hMSCs using the miRNeasy and RNeasy kits (Qiagen, Venlo, The Netherlands), following the methodology established in our prior study [20]. A 1 µg aliquot of the RNA fraction, encompassing miRNAs, from each of the five OS cell lines and hMSCs was biotin-labeled using the Biotin HSR RNA Labeling Kits (Genisphere, Pennsylvania, USA). MiRNA expression profiles were generated using the GeneChip miRNA 3.0 array (Affymetrix, California, USA). For mRNA expression analysis, 1 ng of total RNA from each of the five OS cell lines and hMSCs was utilized to generate double-stranded cDNA through reverse transcription. The 3′ IVT Express Kit (Affymetrix) was employed for *in vitro* generation of biotinylated cRNA. Subsequently, the cRNA probes were hybridized onto the Human Genome U133 Plus 2.0 Array (Affymetrix). Normalized microarray data were analyzed using GeneSpring GX software (Agilent, California, USA), and the list of miRNAs and mRNAs was further filtered to include those displaying a minimum 2-fold change in expression.

### Target prediction of miRNAs

MiRNAs exert their control over gene expression at the post-transcriptional level, orchestrating mRNA degradation and inhibiting translation. This regulatory process is achieved through the establishment of complementary base pairs with their target mRNAs. Of particular significance is the miRNA seed region, spanning nucleotides 2 to 8 in a 5′ to 3′ direction, which exhibits impeccable complementarity with the 3′ UTR of the mRNA [21]. Therefore, many tools, such as TargetScan, analyze the seed area to be a crucial biological component for predicting miRNA targets. In this investigation, TargetScan 8.0 (https://www.targetscan.org/vert_80/), miRTarBase 9.0 (https://mirtarbase.cuhk.edu.cn/), and RNAInter 4.0 (http://www.rnainter.org/) were utilized to search for the matched genes of miR-329-3p, and TCF7L1 was found to be one of its target candidates [22–24].

### RNA-sequencing patient data and bioinformatics analysis

The Cancer Genome Atlas (TCGA) is a groundbreaking collaborative initiative focused on consolidating an extensive repository of cancer-related information [25]. This data is openly accessible, affording scientists worldwide the opportunity to leverage it in their investigations, gaining critical insights into the fundamental mechanisms driving cancer. For the purpose of this study, we accessed a dataset comprising 261 cases of gene expression data, complemented by pertinent clinical information. This comprehensive resource facilitated our in-depth analysis of cancer-associated factors.

### Transfection of mature miRNA and knockdown of target gene using siRNA

OS cells were seeded onto 6-well plates (1 × 10^5^ cells/well) and cultured in complete medium (2 ml/well) devoid of antibiotics for a day prior to transfection. Following the manufacturer’s guidelines, we employed Lipofectamine 2000 (Invitrogen) for transfecting the miR-329-3p mimic (MISSION; Sigma-Aldrich, Massachusetts, USA) or the negative control (NC) miRNA (mirVana; Invitrogen, California, USA) in Opti-MEM (Gibco) reduced serum medium. The transfected cells were extracted and processed for further investigation after 48 h of incubation. As per the protocol, Lipofectamine 2000 was also used to transfect siRNA oligonucleotides targeting TCF7L1 mRNA (Silencer; Ambion, Texas, USA) and siRNA universal negative control (MISSION; Sigma-Aldrich). Cells were then harvested for various analyses. The experiment was conducted in triplicate.

### Reverse transcription quantitative PCR (RT-qPCR)

TRIzol reagent (Invitrogen) was used to lyse and extract total RNA from transfected cells and subsequently converted to cDNA. Real-time PCR (qPCR) analysis was performed on a LightCycler 96 System (Roche, Rotkreuz, Switzerland) to quantify the expression levels of TCF7L1 and miR-329-3p. The 2^−∆∆Ct^ method was employed for relative quantification. Specifically, the primers used were as follows: TCF7L1-forward, 5′-GAAGAAGCCTCTGAATGCCT-3′ and TCF7L1-reverse, 5′-GGGACAGCTGCTTTTCTCTC-3′; GAPDH-forward, 5′-TGCCTCCTGCACCACCAACT-3′ and GAPDH-reverse, 5′-CCCGTTCAGCTCAGGGATGA-3′. U6snRNA-forward, 5′-GCTTCGGCAGCACATATACTAAAAT-3′ and U6snRNA-reverse, 5′-CGCTTCACGAATTTGGCGTGTCAT-3′.

### Western blot analysis

Total cellular protein (15 µg) was separated on an 8.5% Tris-HCl Criterion 12-well gel (Mini-PROTEAN; Bio-Rad, California, USA) at 200V for 30 min. Protein transfer from gels to PVDF membranes was carried out using a semi-dry system (Bio-Rad) set at 250 V, 0.1 A for 30 min. Rabbit source primary antibodies (×1000) against TCF7L1 (#2883), PARP (#9532), c-PARP (#5625), β-catenin (#8480), Cyclin D1 (#55506), c-Myc (#5605) and GAPDH (#5174) were obtained from Cell Signal Technology (Tokyo, Japan). Blots were developed using Chemi-Lumi One Super (Nacalai Tesque, Kyoto, Japan) and visualized with HRP-Goat Anti-Rabbit IgG (JIRL, Pennsylvania, USA) using the ImageQuant 800 system (Cytiva, Massachusetts, USA). Protein expression was quantified using NIH’s ImageJ software. There were three duplicates of each experiment.

### Dual luciferase assay

The dual luciferase assay was used to verify whether TCF7L1 was a direct target of miR-329-3p. The ligation sites were cleaved by Dra I and Xba I, and the TCF7L1 sequence (Takara) carrying the complementary site of miR-329-3p was introduced into the pmirGLO luciferase reporter vector (Promega, Wisconsin, USA) by DNA Ligation Kit (Takara) to establish the wild type of TCF7L1 (TCF7L1-Wt) luciferase reporter plasmid and cloned using *E. coli* JM109 competent cell. Similarly, a mutant (TCF7L1-Mut) luciferase reporter plasmid of TCF7L1 was generated by mutating the combinatorial site of miR-329-3p and cloned. The oligonucleotide sequences are provided below, with the seed region of the miR-329-3p targeting site indicated by underlining. Additionally, the complementary sequence of the seed region in the antisense primer is highlighted in bold.

Wild type sense: 5′-AAACTAGCGGCCGCTAGTTCACATGCTTCTTCTTCTGTGTGTATT-3′.

Wild type anti-sense: 5′-CTAGAAT**ACACAC**AGAAGAAGAAGCATGTGAACTAGCGGCCGCTAGTTT-3′.

Mutant sense: 5′-AAACTAGCGGCCGCTAGTTCACATGCTTCTTCTTCTCACACAATT-3′.

Mutant anti-sense: 5′-CTAGAAT**TGTGTG**AGAAGAAGAAGCATGTGAACTAGCGGCCGCTAGTTT-3′.

Following the co-transfection of the miR-329-3p mimic and miRNA-NC with the previously mentioned luciferase reporter plasmids, cells were allowed to incubate for 48 h. Subsequently, the cells were lysed utilizing the Dual-Glo Luciferase Assay System (Promega). The luciferase activity was then quantified using the GloMax-Multi+ Detection System (Promega). This assay provided a quantitative measure of luciferase expression, allowing for the assessment of miR-329-3p’s impact on the target gene. There were three duplicates of each experiment.

### Cell proliferation assay

After seeding at a density of 1 × 10^5^ cells per well in 6-well plates, the cells were subjected to transfection with one of the following: miR-329-3p mimic, negative control miRNA, TCF7L1-siRNA, or negative control siRNA. The cells were then incubated in DMEM from Gibco, an antibiotic-free cell culture medium. Cell counts were obtained after 48 h of incubation using the TC10 Automated Cell Counter (Bio-Rad). The TC10 system employs trypan blue staining to distinguish between live and dead cells, ensuring accurate cell count determination. There were three duplicates of each experiment. The cell count data was then subjected to statistical analysis to evaluate the impact of miR-329-3p and TCF7L1 modulation on cell proliferation.

### Flow cytometry

MG-63 cells were transfected with miR-329-3p mimic, negative control miRNA, and TCF7L1-siRNA after being plated in a 6-well plate at a density of 1 × 10^5^ cells per well. The samples were trypsinized and overnight fixed in cold 70% ethanol after 48 h of transfection. Propidium iodide was used to label the cells and cell cycle distribution was assessed using an LSRFortessa flow cytometer (BD, New Jersey, USA), with data analyzed using Flowjo v10 software (BD). This process enabled a comprehensive examination of the cell cycle dynamics in response to miR-329-3p mimic, negative control miRNA, and TCF7L1-siRNA transfection. The experiment was conducted in triplicate to ensure reliable and consistent results.

### Immunofluorescence analysis

To scrutinize the effects of miR-329-3p and TCF7L1-siRNA transfection on the division, migration, and apoptosis of osteosarcoma cells, alterations in cell morphology were examined through confocal microscopy (LSM 710, ZEISS, Oberkochen, Germany). Initially, cells were fixed in a 4% paraformaldehyde solution for 30 min to preserve cellular structures. Following fixation, transfected cells were permeabilized with 0.1% Triton X-100 for 20 min. Subsequently, for F-actin visualization, cells were incubated overnight at 4°C with Alexa Fluor 488 Phalloidin (diluted at 1:100, Invitrogen) for immunofluorescence staining. The nucleus was counterstained using ProLong Diamond Antifade Mountant with DAPI (Invitrogen). This detailed protocol enabled a comprehensive evaluation of cellular morphology, providing crucial insights into the influence of miR-329-3p and TCF7L1-siRNA transfection on cell behavior.

### Xenotransplantation experiment

A total of 28 BALB/c nu/nu mice, each at 5 weeks of age, were acquired from the Kudo Lab located in Tosu, Japan. Following a stipulated quarantine period, the mice were accommodated in a controlled environment free of pathogens. This environment adhered to a consistent 12-h light/12-h dark cycle, and the mice were provided with a standard sterilized pellet diet, along with unrestricted access to water. The health of mice was continuously monitored during weekdays and twice daily on weekends and holidays. The establishment of the experimental tumor heterograft model entailed the subcutaneous injection of 2 × 10^6^ miR-329-3p-transfected cells in 100 μl of sterile saline into the unilateral subgluteal region of immunodeficient nude mice. The injection site was meticulously selected, and a fine-gauge needle was employed to ensure precise delivery of the cell suspension. There were four groups created: (1) untreated control (*n* = 5); (2) negative control-miRNA transfected group (*n* = 5); (3) miR-329-3p transfected group (*n* = 5); and (4) TCF7L1-siRNA transfected group (*n* = 5). Six weeks post cell injection, all animals were euthanized under standard conditions. The xenograft tumors in nude mice were measured for length (L) and width (W) using calipers in two dimensions parallel to each other, and the primary tumor volume (V) was calculated using the formula V = 1/2 (L × W^2^), providing precise data on tumor dimensions for further analysis [26]. The animal experiment protocol was approved by the Animal Experiment Ethics Review Committee of Oita University, Japan, and all animal experiments were performed in accordance with the guidelines for animal experimentation prescribed by the Oita University Faculty of Medicine, and were reviewed and supervised by the Oita University Graduate School of Medical Science.

### Immunohistochemistry (IHC)

The xenograft tumor tissue was meticulously fixed with a 10% neutral buffered formalin solution, followed by a rigorous dehydration process using graded ethanol, culminating in a transition through xylene. Subsequently, the tissue was judiciously embedded in paraffin wax and expertly sectioned to a thickness of 4 micrometers. Post deparaffinization and rehydration, the tissue underwent a critical antigen retrieval step, involving a precisely timed 20-min incubation at 99°C with 10 mM citrate buffer (pH 6.0). To minimize nonspecific antibody binding, the sections were meticulously treated with One Histo blocking solution (Takara). To further enhance specificity, avidin from egg white (Nacalai Tesque) and D-Biotin (Nacalai Tesque) were employed to quench any potential endogenous biotin interference. In addition, endogenous peroxidase activity was effectively mitigated by immersing the sections in a 0.3% hydrogen peroxide solution. The primary antibodies (Ki-67, #9027; CST; c-Myc, ab32072; Abcam, Cambridge, UK; TCF3, ab69999; Abcam) were appropriately diluted and added dropwise onto the slides. Subsequently, the slides were placed in a humidified environment and left to incubate overnight at 4°C. Following this, the slides were treated with Biotin-SP-conjugated antibodies (Jackson ImmunoResearch). The antigen-antibody complexes were detected using the Strept ABC Peroxidase Kit from Nacalai tesque, and visualized through development with diaminobenzidine using the Peroxidase Stain DAB Kit (Nacalai tesque). To enhance contrast, sections were counterstained with hematoxylin. A fluorescent microscope (KEYENCE, Osaka, Japan) was used to take the images. The staining outcome was calculated by combining the percentage of tumor cells that stained positively with the degree of positive staining [27].

### Statistical analysis

The statistical analysis was conducted using GraphPad 8.0 software (California, USA). Continuous variables underwent analysis through a two-tailed Student’s *t*-test. The comparison of statistical differences between two groups utilized the Wilcoxon test, while differences among three groups were assessed with the Kruskal-Wallis test. In cases involving more than three groups, non-repeated measures analysis of variance (ANOVA) followed by the Scheffe test was applied. The results were presented as mean ± SD, and a *p*-value below 0.05 was considered statistically significant.

## Results

### Whole-gene microarray analysis indicates negative regulatory between miR-329-3p and TCF7L1 in OS cells

**Figure 1:**
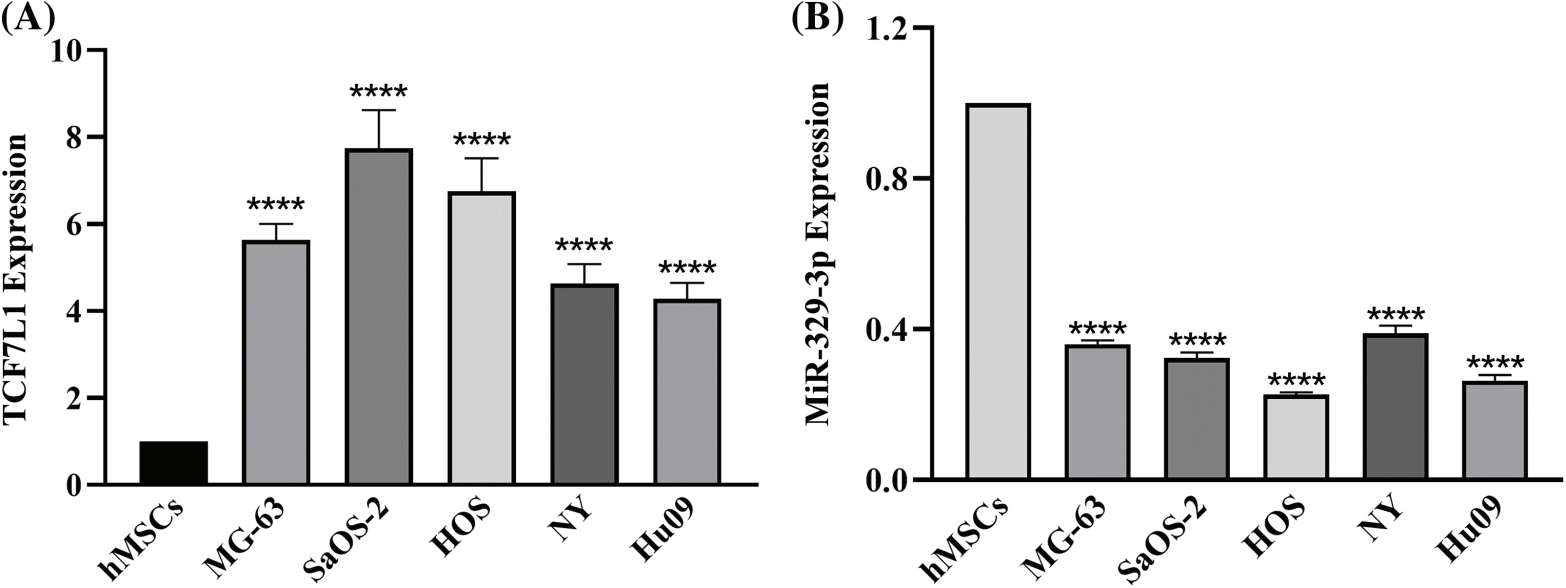
qRT-PCR analysis of TCF7L1 and miR-329-3p expression in hMSCs and OS Cell Lines. (A) Expression levels of TCF7L1 were assessed by qRT-PCR in hMSCs and five OS cell lines, namely MG-63, SaOS-2, HOS, NY, and Hu09. (B) The expression profile of miR-329-3p was examined using qRT-PCR in hMSCs and the OS cell lines MG-63, SaOS-2, HOS, NY, and Hu09. ****p < 0.0001.

When compared to the hMSCs, the expression levels of 435 miRNAs in the five OS cell lines indicated a fold change of >2.0, according to prior results of our genome-wide miRNA expression profiling [20]. The expression of miR-329-3p was lower in the OS cell lines than in the hMSCs by −2.55 to −4.38 fold (Suppl. Fig. S1). Likewise, cDNA array analysis revealed significant disparities in the expression of 761 mRNAs between the five OS cell lines and hMSCs. In comparison to hMSCs, OS cell lines expressed TCF7L1 at a level that was 3.27 to 9.97 times greater (Suppl. Fig. S2). Subsequently, we employed qPCR to assess the expression of TCF7L1 and miR-329-3p in hMSCs and five OS cell lines, including MG-63, SaOS-2, HOS, NY, and Hu09. Comparative to hMSCs, TCF7L1 expression significantly increased, while miR-329-3p expression significantly decreased in OS cells (Figs. 1A and 1B). The qPCR findings concurred with the microarray data, affirming a negative correlation between TCF7L1 and miR-329-3p in OS cells.

### TCGA data showing the reciprocal correlation between miR-329-3p and TCF7L1 and its impact on prognosis

Analysis of TCGA data revealed a reciprocal correlation between miR-329-3p and TCF7L1, along with its impact on prognosis. In sarcoma tissues, TCF7L1 expression and miR-329-3p levels demonstrated a significant inverse relationship (Suppl. Figs. S3A and S3B). Moreover, miR-329-3p did not exhibit a correlation with prognosis (Suppl. Fig. S3C). However, elevated mRNA expression of TCF7L1 in sarcoma patients was associated with a poorer overall survival outcome, and high hazard ratios indicated that TCF7L1 acted as a risk factor in sarcoma (Suppl. Fig. S3D). These findings suggest that the repressive regulation of TCF7L1 by miR-329-3p holds significant prognostic value for sarcoma patients.

### MiR-329-3p targets TCF7L1 mRNA directly

**Figure 2:**
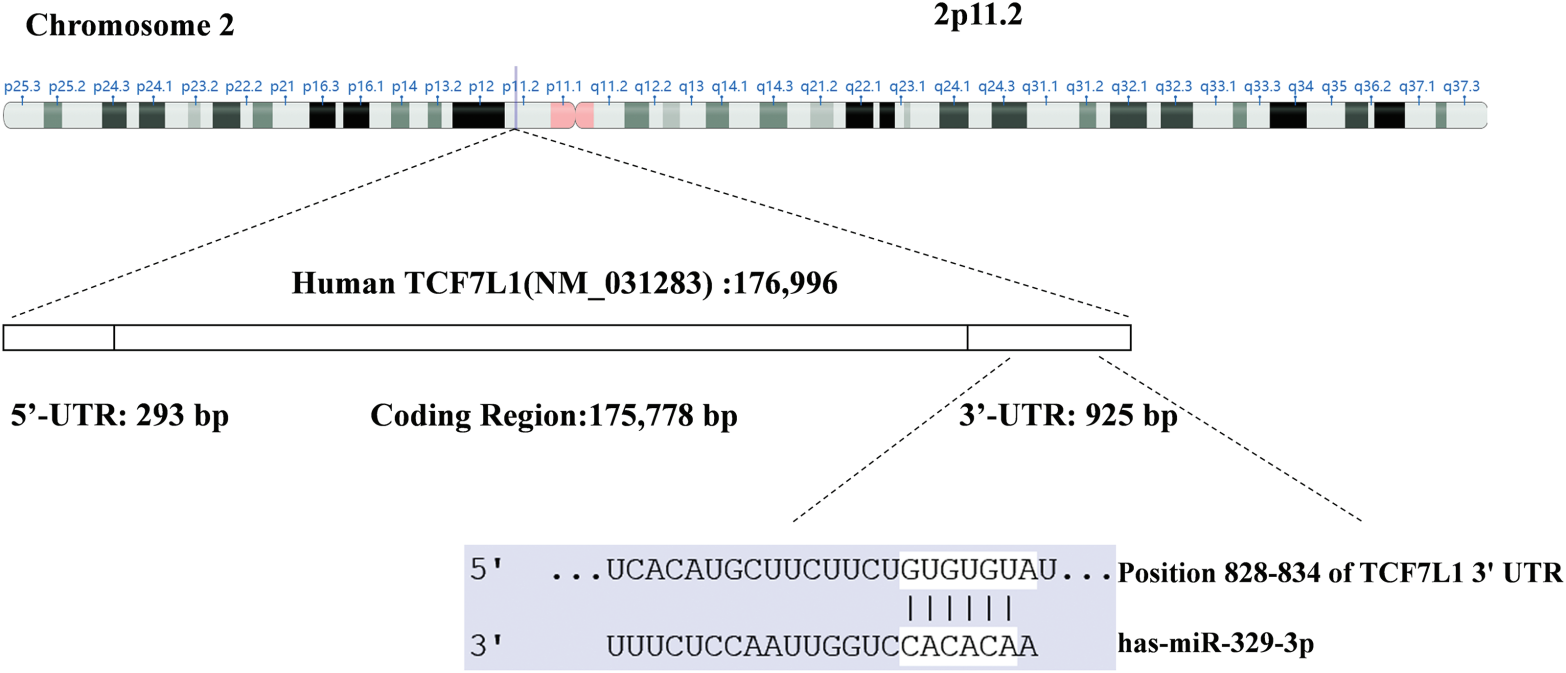
Through the utilization of TargetScan and the miRanda algorithm, a region within the 3′-UTR of human *TCF7L1* exhibiting complementarity to the seed region of miR-329-3p was identified.

**Figure 3:**
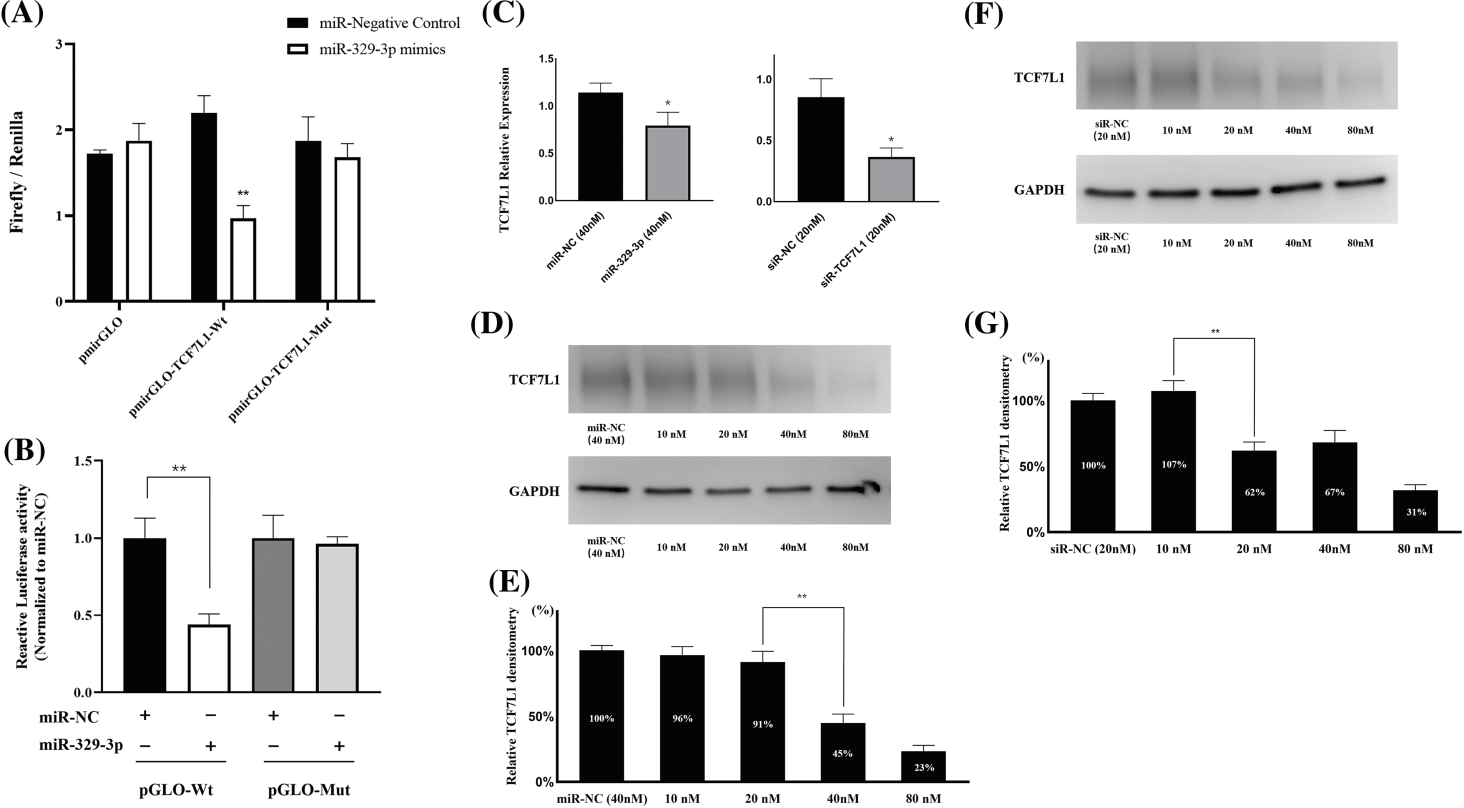
MiR-329-3p directly targets TCF7L1 mRNA. (A) The dual-luciferase assay was employed to establish the targeting interaction between miR-329-3p and TCF7L1, providing evidence for their direct association. (B) Co-transfection of MG-63 cells with miR-329-3p mimics, NC, and either pmirGLO-TCF7L1-Wt or pmirGLO-TCF7L1-Mut. Using the dual-luciferase test, luciferase activity was found 48 h after transfection. (C) qRT-PCR was used to determine the levels of mRNA produced by TCF7L1 following the transfection of miR-329-3p, TCF7L1-siR, or negative control oligonucleotides. (D) Decreased TCF7L1 was found using a Western blot analysis in MG-63 cells that had been transfected with miR-329-3p. (E) Quantification of the TCF7L1 protein using densitometry following miR-329-3p transfection. (F) Decreased TCF7L1 was found using a Western blot analysis in MG-63 cells that had been transfected with TCF7L1-siRNA. (G) Quantification of the TCF7L1 protein using densitometry following TCF7L1-siRNA transfection. *p < 0.05, **p < 0.01.

Utilizing TargetScan and miRanda algorithms, we identified a total of 190 mRNA targets for miR-329-3p (Suppl. Table S1). Notably, we discovered a region within the 3′-UTR of human TCF7L1 mRNA that exhibits complementarity to the seed sequence of miR-329-3p (Fig. 2 and Suppl. Fig. S4). This interaction theoretically resulted in differential expression of TCF7L1. In MG-63 cells, when miR-329-3p mimics were transfected, the 3′-UTR of TCF7L1 mRNA was validated as the binding site for miR-329-3p through a dual luciferase assay. The data demonstrated a significant reduction in luciferase activity induced by TCF7L1 3′-UTR in the miR-329-3p mimic group compared to the control group. Moreover, upon mutating the presumed binding site, it was observed that the inhibitory effect of miR-329-3p was attenuated. This finding strongly suggests a direct targeting of TCF7L1 mRNA by miR-329-3p (Figs. 3A and 3B). The RT-qPCR results displayed a marked decrease in TCF7L1 expression in cells transfected with either miR-329-3p or TCF7L1-siRNA, in contrast to those transfected with negative control oligonucleotides (Fig. 3C). Western blotting further confirmed the significant reduction in TCF7L1 protein expression in miR-329-3p-transfected cells compared to untreated or NC oligo-transfected cells (Fig. 3D). Specifically, miR-329-3p-transfected cells exhibited a 45% reduction in TCF7L1 protein expression compared to control cells (Fig. 3E). The effectiveness of the transfection technique was further corroborated through the transfection of siRNA targeting TCF7L1, which led to a substantial 62% reduction in TCF7L1 expression compared to control cells (Figs. 3F and 3G). This collectively indicates that miR-329-3p directly targets TCF7L1 mRNA.

### OS cell proliferation is inhibited by miR-329-3p and TCF7L1-siRNA

**Figure 4:**
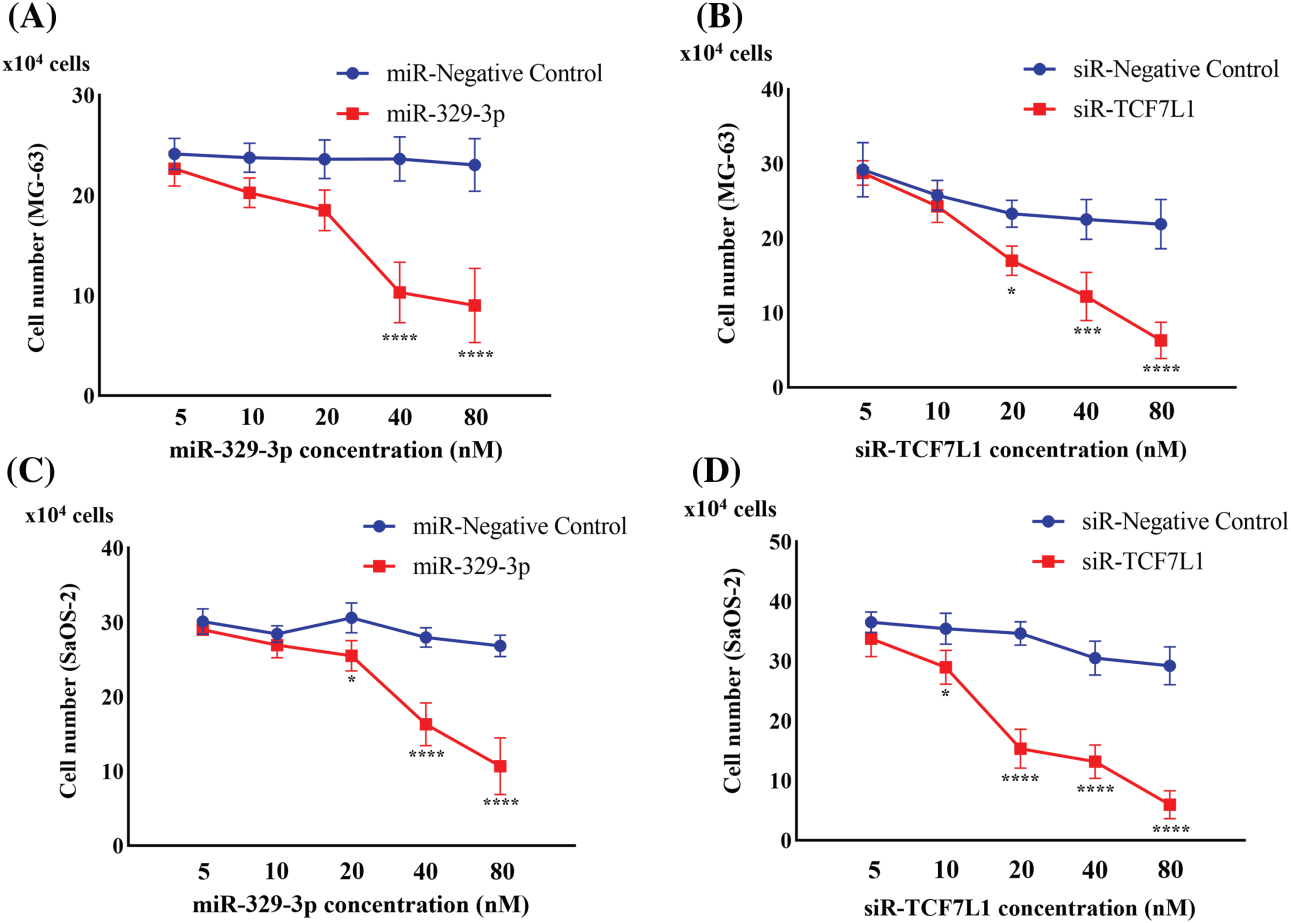
Cell proliferation assay evaluating the anti-proliferation effect. (A) Cell counts were obtained 48 h after the introduction of miR-329-3p mimics or negative control (NC) into MG-63 cells. (B) Cell counts were obtained 48 h after the introduction of TCF7L1-siRNA or NC into MG-63 cells. (C) Cell counts were obtained 48 h after the introduction of miR-329-3p mimics or NC into SaOS-2 cells. (D) Cell counts were obtained 48 h after the introduction of TCF7L1-siRNA or NC into SaOS-2 cells. Asterisks (*) denote the significance levels of the *p*-value, where *p < 0.05, ***p < 0.001, and ****p < 0.0001.

Upon transfection with miR-329-3p and TCF7L1-siRNA, a pronounced inhibition of cell proliferation was observed in both MG-63 and SaOS-2 cells. To delve deeper, we employed a range of transfection concentrations, varying from 5 to 80 nM. The proliferation curves of the control, miR-NC, and siR-NC groups displayed minimal fluctuations as drug concentration increased. Noteworthy observations were made in MG-63 cells; the miR-329-3p group exhibited no discernible alterations at concentrations up to 20 nM, while substantial changes became evident at higher concentrations, resulting in a significant reduction in cell viability (Fig. 4A). Similarly, SaOS-2 cells transfected with miR-329-3p displayed no substantial shifts at concentrations below 10 nM, but noteworthy changes were observed at higher concentrations, coinciding with an increase in cell death (Fig. 4C). In the TCF7L1-siRNA group, MG-63 cells demonstrated no significant changes at concentrations of 5 and 10 nM, but substantial alterations occurred at concentrations above 20 nM, leading to a substantial decline in cell viability (Fig. 4B). Likewise, SaOS-2 cells exhibited no significant variations when the concentration was below 5 nM, but significant changes occurred at concentrations above 10 nM, accompanied by a rise in cell death (Fig. 4D).

### Induction of cell cycle arrest in G0/G1 by miR-329-3p

**Figure 5:**
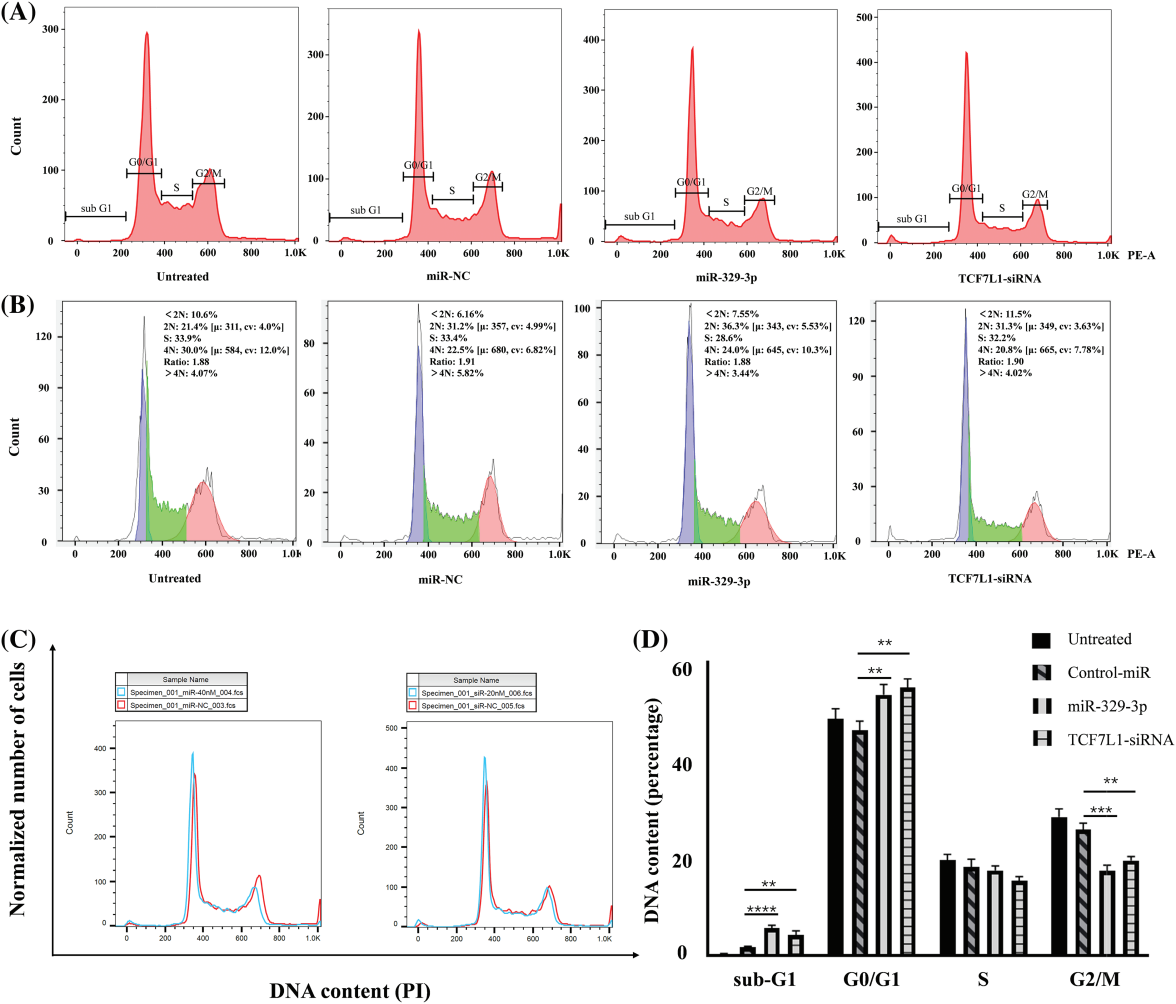
The impact of miR-329-3p and TCF7L1-siRNA on cell cycle distribution in MG-63 cells. (A) Following transfection for 48 h, MG-63 cells were subjected to propidium iodide staining and analyzed using flow cytometry. (B) Comparative assessment of haploid and diploid cell distribution. (C) Comparison of cells in the G0/G1 and G2/M phases. (D) Histogram presenting the quantitative percentage of DNA content in each cell cycle phase. One-way ANOVA was utilized for significance determination of each group, with Tukey’s test applied for variance correction. **p < 0.01, ***p < 0.001, ****p < 0.0001.

Given the significant inhibitory impact of miR-329-3p introduction on the proliferation of OS cells and the classification of TCF7L1, a miR-329-3p target, within the transcription factor family, our investigation extended to explore the regulatory influence of miR-329-3p on the cell cycle. The data demonstrates that compared with untreated or negative control cells, the overexpression of miR-329-3p and knockdown of TCF7L1 lead to OS cells progressing from the G0/G1 phase to subsequent stages of DNA synthesis and cell division (S phases and G2/M phases) being hindered. A substantial number of cells are arrested in the resting phase, failing to enter the cell division state (Fig. 5A). In the cell lines transfected with miR-329-3p and TCF7L1-siRNA, the tetraploid cell population in the division phase and the cell population in the DNA synthesis and replication stage are significantly reduced. Conversely, the proportion of diploid cell populations with normal DNA content increases (Fig. 5B).

Moreover, in addition to the observed alterations in G0/G1 and G2/M phases, we investigated the sub-G1 phase, which typically represents cells undergoing apoptosis or those with fragmented DNA. Our data revealed a significant increase in the sub-G1 population in cells transfected with miR-329-3p and TCF7L1-siRNA, indicating that the targeting of TCF7L1 by miR-329-3p not only leads to cell cycle arrest but also promotes apoptosis of the cells (Fig. 5C). The bar graph depicted for each cell cycle underscores the significant impact of miR-329-3p on OS, showcasing its pivotal role in governing cell cycle process related to proliferation and survival (Fig. 5D).

### MiR-329-3p hinders cell proliferation through Wnt/β-catenin pathway

**Figure 6:**
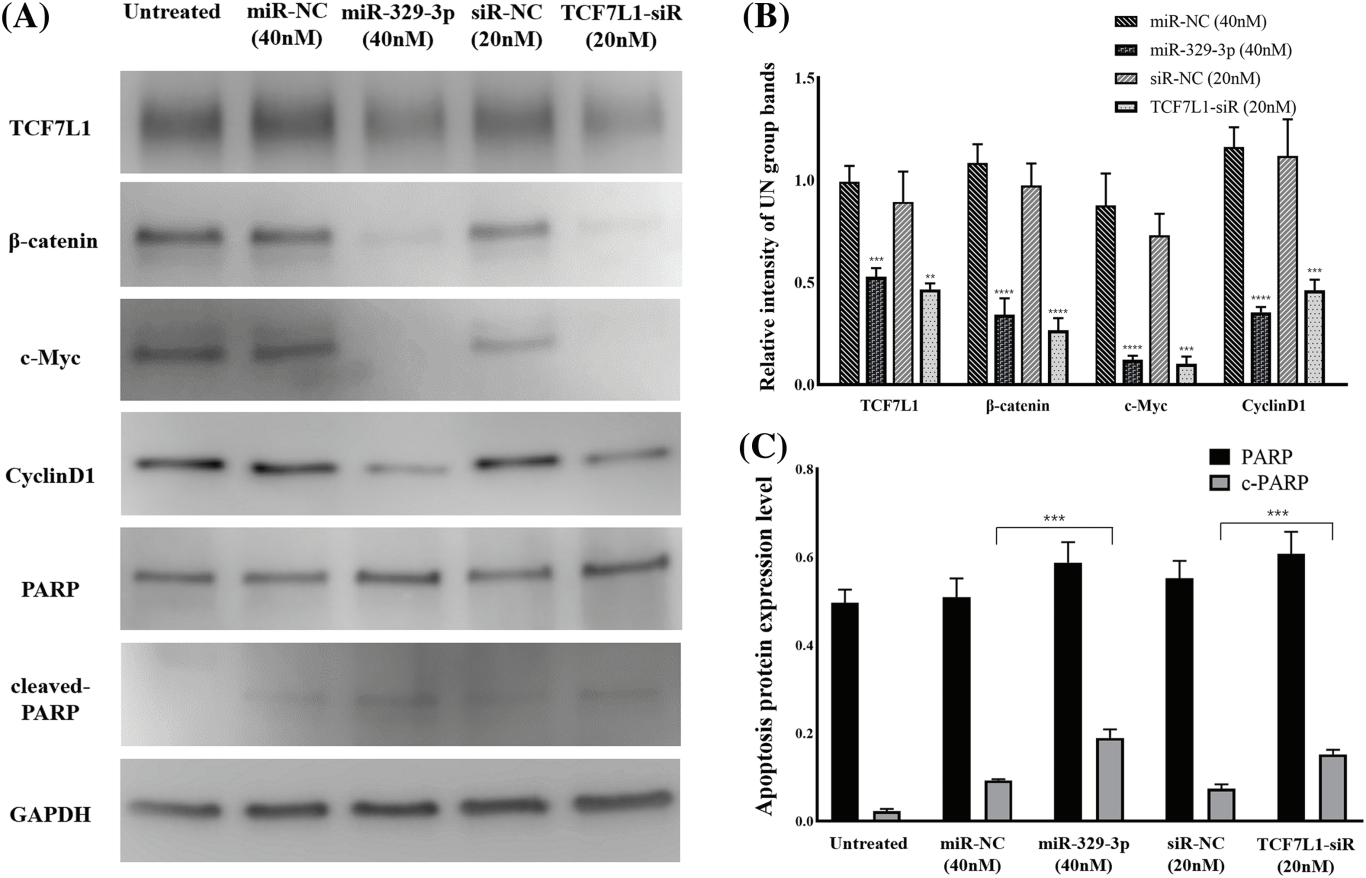
TCF7L1 was investigated for its association with Wnt/-catenin pathway, cell cycle, and apoptotic components using Western blotting. (A) Effect of miR-329-3p and TCF7L1-siRNA on TCF7L1 protein expression and its up/downstream factors in MG-63 cells after 48 h of transfection. (B) Quantification of protein expression levels related to the Wnt/β-catenin pathway. (C) Protein expression levels associated with apoptosis were measured using densitometry. **p < 0.01, ***p < 0.001, ****p < 0.0001.

MiR-329-3p suppressed the mRNA expression of TCF7L1. We examined the upstream β-catenin of TCF7L1 and downstream factors (c-Myc and Cyclin D1) associated with cell proliferation and cell cycle in the Wnt/β-catenin pathway. Western blot analysis revealed that TCF7L1 suppression by miR-329-3p mimics and TCF7L1-siRNA transfection inhibited Wnt/β-catenin pathway components β-catenin, c-Myc, and Cyclin D1 (Figs. 6A and 6B). In addition, the cellular expression levels of PARP and its cleaved product, cleaved-PARP, a marker of caspase-mediated apoptosis, were markedly elevated in cells transfected with miR-329-3p mimics and TCF7L1-siRNA compared to the control cells (Fig. 6C). Based on these findings, it appears that miR-329-3p can suppress OS cell proliferation via the Wnt/β-catenin pathway.

### Disorganization of actin microfilaments accompanying apoptosis

**Figure 7:**
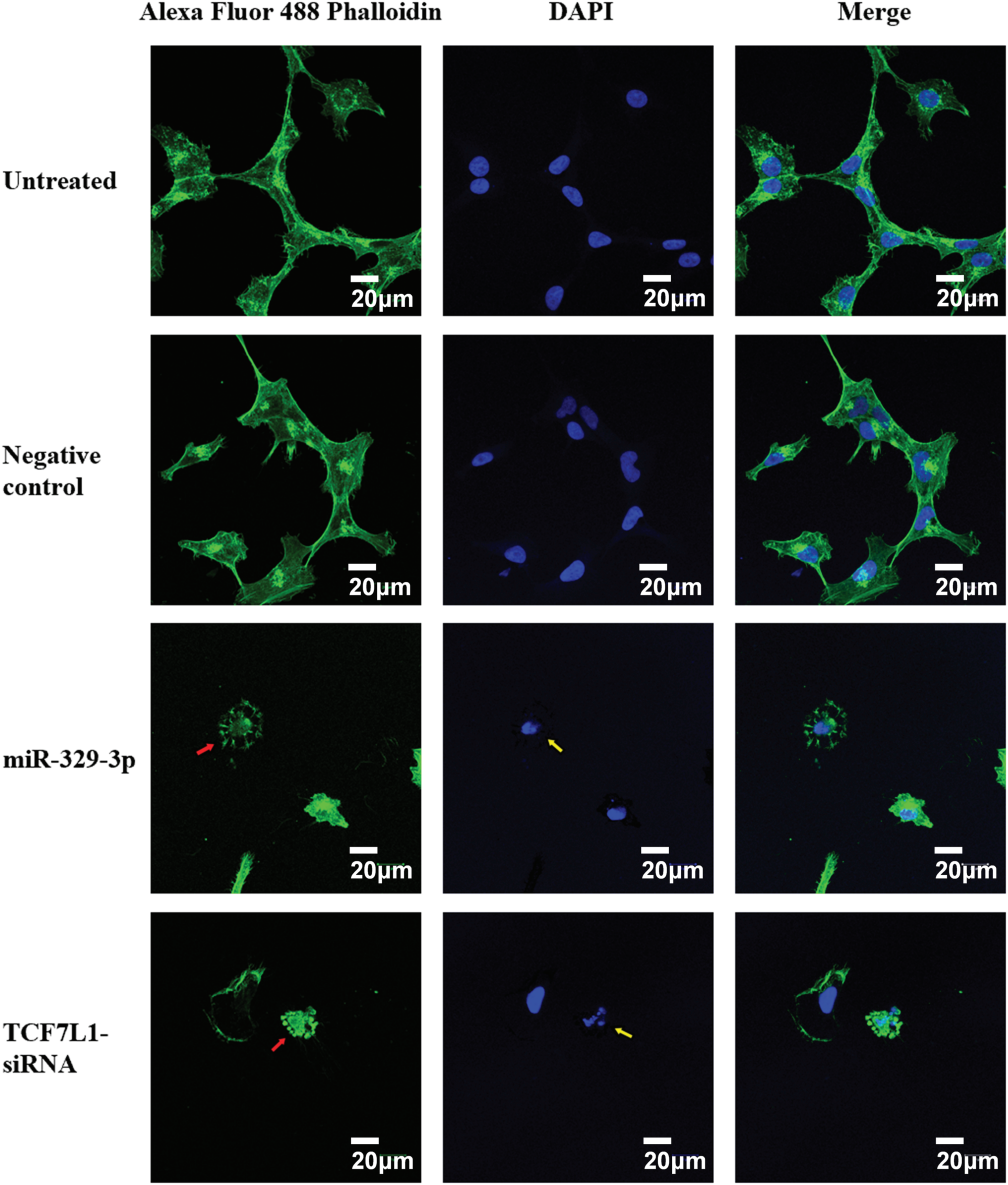
Cell apoptosis and microfilament alterations following miR-329-3p or TCF7L-siRNA treatment (phalloidin staining and DAPI counterstaining). Laser scanning confocal microscopy at a magnification of 400× was used to view the cells. Scale bar = 20 μm. Green, F-actin; blue, nucleus. Following miR-329-3p or TCF7L1 treatment, microfilament disintegration is represented by red arrows, and nuclear condensation and apoptotic bodies are represented by yellow arrows.

We studied cytoskeletal dynamics to investigate the cellular mechanisms by which miR-329-3p and TCF7L1 siRNA induce OS apoptosis. The outcomes demonstrated that the MG-63 cells treated with miR-329-3p and TCF7L1 siRNA shrank, and many of these cells were suspended in the medium. Therefore, we performed F-actin staining with an Alexa Fluor 488-labeled Phalloidin probe in MG-63 cells. The treated group had significantly fewer cells in the field of view, more unevenly distributed F-actin in the cytoplasm, and brighter fluorophores than those in the untreated or miR-NC transfection groups. These findings provide evidence that the introduction of miR-329-3p and TCF7L1 siRNA led to the disassembly of actin filament structures in the MG-63 cells. DAPI labeling was used to see the cell nuclei and apoptotic bodies (condensed and fragmented nuclei). The MG-63 cells in the control group developed healthily, exhibited constant brightness, and had uniformly distributed chromatin throughout their nuclei. But the cells in the treated groups exhibited pyknotic nuclear chromatin, which was frequently marginated along the nuclear border, giving the appearance of half-moon-like nuclei. Occasionally, characteristic apoptotic structures were also formed (Fig. 7).

### Xenograft tumor inhibition by miR-329-3p and TCF7L1-siRNA

**Figure 8:**
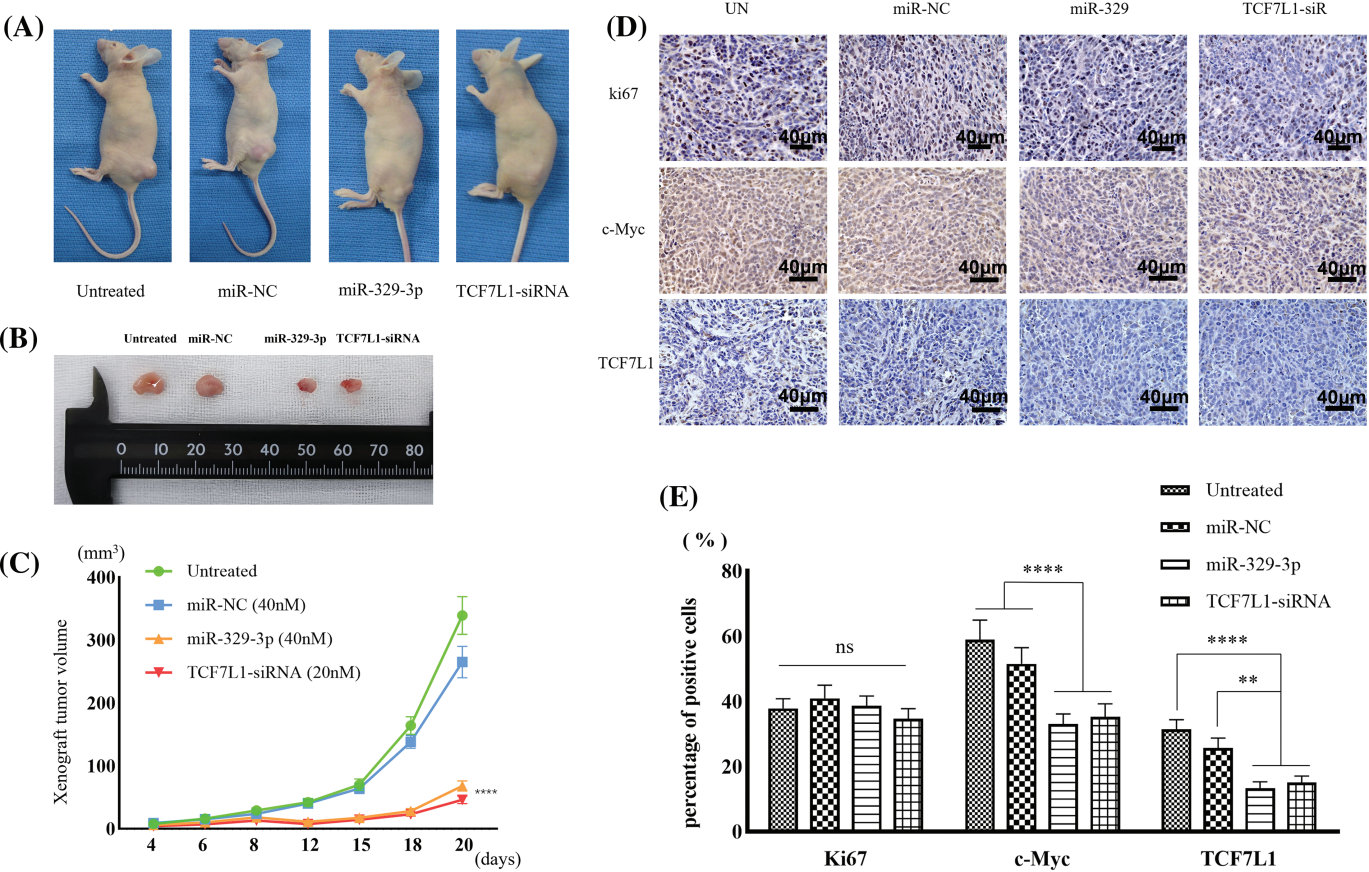
Xenograft tumors inhibited by miR-329-3p and TCF7L1-siRNA. (A) Photographs depicting the xenograft tumor. (B) Comparison of xenograft tumor size. (C) Following the inoculation of tumor cells, tumor volumes were measured at the indicated times. (D) TCF7L1, c-Myc, and ki67 expression changes in the xenograft tumor transfected with miR-329-3p and TCF7L1 siRNA. Magnification at origin: 200×; Scale bars = 40 μm. (E) Percentages of cells expressing ki67, c-Myc, and TCF7L1 in each group. One-way analysis of variance was employed to establish the significance of each group (n = 5). Tukey’s test was applied to correct for variance. Asterisks (*) denote the significance levels of the *p*-value, ns: *p* ≥ 0.05, **p < 0.01, ****p < 0.0001.

Mimics of miR-329-3p or TCF7L1-siRNA were introduced into MG-63 cells, which resulted in a reduction in the formation of subcutaneous xenograft tumors in nude mice (Figs. 8A and 8B). The tumor sizes in the mice inoculated with miR-329-3p-transfected MG-63 cells (68.4 ± 8.3 cells/mm^3^) or TCF7L1-siRNA-transfected MG-63 cells (46.5 ± 6.5 cells/mm^3^) were significantly smaller than those transfected with untreated (339.2 ± 31.7 cells/mm^3^) and NC-miRNA-transfected cells (266.3 ± 14.6 cells/mm^3^) (Fig. 8C). Based on these findings, it appears that miR-329-3p is able to prevent the proliferation of OS cells *in vivo*.

In comparison to the Ki67-positive control group, immunohistochemical examination revealed that transfection with miR-329-3p and TCF7L1-siRNA resulted in a decrease in the expression of TCF7L1 and c-Myc in xenografted tumors (Fig. 8D). The percentage of cells expressing TCF7L1 significantly decreased in mice inoculated with miR-329-3p- (13.3% ± 1.6%) or TCF7L1-siRNA-transfected cells (15.1% ± 2.4%) compared to untreated (31.4% ± 3.7%) or miR-NC-transfected cells (25.7% ± 3.0%). The percentage of cells expressing c-Myc was significantly lower in xenograft tumors inoculated with miR-329-3p- (33.1% ± 3.8%) or TCF7L1-siRNA-transfected cells (35.2% ± 4.6%) than in those inoculated with untreated (58.9% ± 6.3%) or miR-NC-transfected cells (51.5% ± 5.1%) (Fig. 8E).

**Figure 9:**
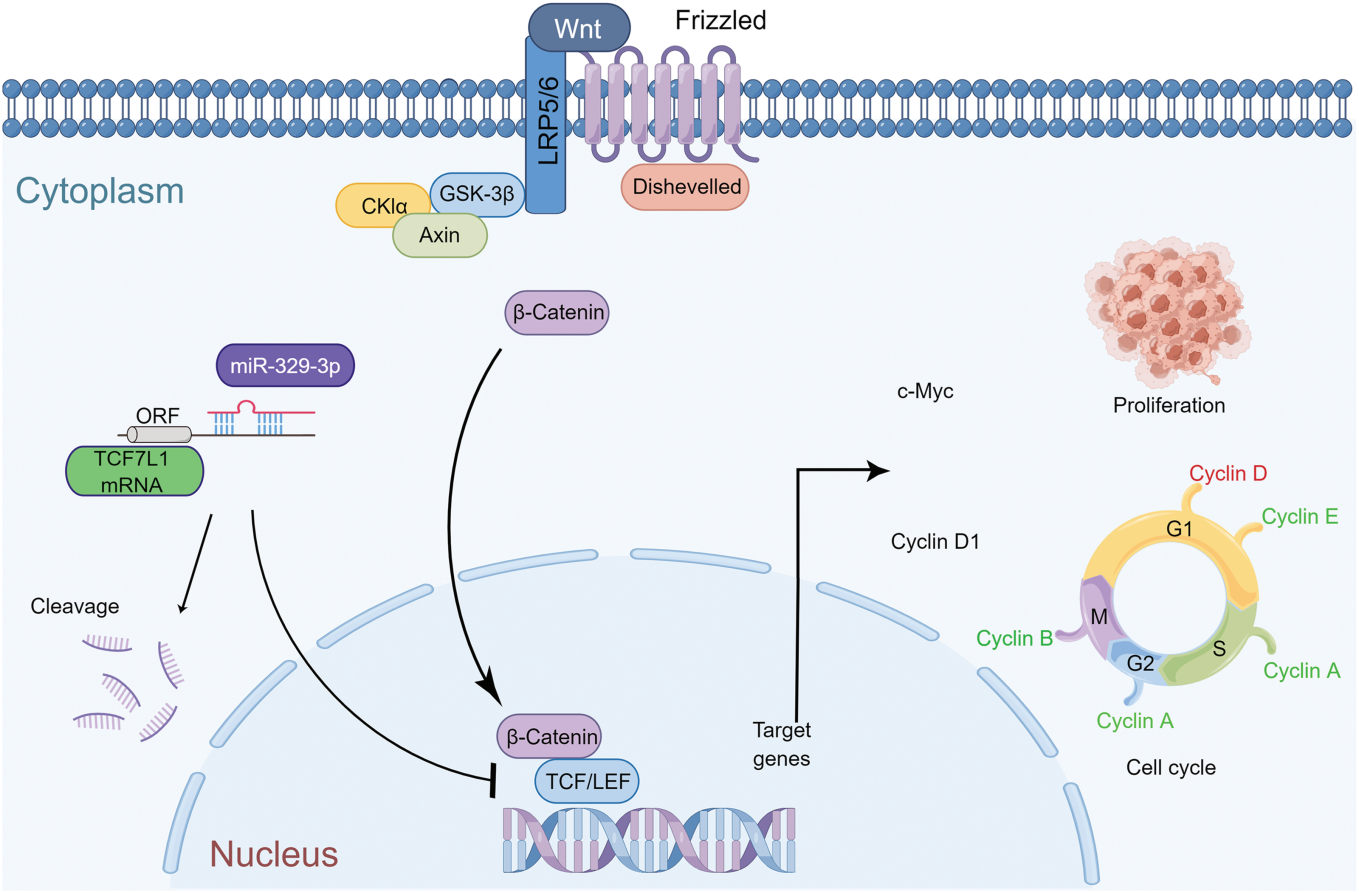
Schematic representation of miR-329-3p-mediated inhibition of human osteosarcoma cells via the Wnt/β-catenin pathway. MiR-329-3p exerts inhibitory effects on osteosarcoma cell proliferation and induces cell cycle arrest by targeting TCF7L1.

In summary, miR-329-3p plays a significant role in inhibiting the growth of osteosarcoma *in vivo*. The mechanism diagram illustrates how miR-329-3p and TCF7L1 regulate the proliferation and cell cycle of OS cells. MiR-329-3p directly targets TCF7L1, leading to the inhibition of TCF/LEF downstream target factors in the Wnt/β-catenin pathway, such as c-Myc and CyclinD1 (Fig. 9).

## Discussion

Improving the survival of patients with OS remains challenging despite advancements in the treatment of this disease [28]. The “targeted therapy” era, which is based on the genomes and proteomics of tumor cells, has officially arrived [29]. Genomic examination of tumor tissue has uncovered possible therapeutic avenues for a wide variety of cancers [30–32]. Microarray technology is a straightforward and convenient method for identifying gene expression alterations associated with disease progression, providing beneficial insights into the diagnosis, treatment, and prognosis of OS.

In this study, we conducted genome-wide microRNA and cDNA array analyses on OS cells, aiming to uncover regulatory connections between miRNA and mRNA relevant to the disease. Cluster analysis of the results highlighted a significant decrease in the expression of miR-329-3p in osteosarcoma cell lines. Previous research has underscored the cancer-specific effects of miR-329-3p, functionally, a recognized tumor suppressor miRNA, plays a crucial role in regulating the cell cycle and curbing tumor characteristics like invasion, growth, differentiation, and migration. Notably, this miRNA has been documented to be downregulated across various cancer types, including OS [11,12,33]. Its targets span a wide spectrum, from the USP22-Wnt/β-catenin pathway in liver cancer cells to MAPK1 in cervical cancer cells [13,34]. It is noteworthy that miR-329-3p also exhibits effects on healthy cells, as demonstrated by its expression pattern during neural stem cell differentiation and its response to exercise intervention in adipose tissue [35,36].

Considering the expression of miR-329-3p was completely linearly downregulated in five OS cell lines (MG-63, SaOS-2, HOS, NY and Hu09), we conducted cDNA array genome-wide mRNA profiling to identify potential targets of miR-329-3p in OS cells. According to the data obtained from the cDNA arrays, the level of mRNA expression of *TCF7L1* was shown to be higher in all of the OS cell lines compared to that of hMSCs. In spite of the fact that miR-329-3p is capable of targeting a variety of genes, including *CD44, E2F1, CREB1*, and *BCL2*, the focus of the present investigation was on determining the connection between miR-329-3p and TCF7L1. The ability of miR-329-3p to bind to the 3′-UTR of TCF7L1 was discovered through computational study employing a number of different approaches [22–24]. We performed a dual-luciferase analysis to verify whether miR-329-3p directly targets *TCF7L1* mRNA. The predicted binding sites of *TCF7L1* 3′-UTR and its mutated version were subcloned downstream of the luciferase gene and transfected along with chemically synthesized miR-329-3p mimics. The fact that luciferase activity was silenced only with the wild-type UTR, but not with the mutated version, confirms that miR-329-3p specifically binds to *TCF7L1* mRNA and negatively regulates gene expression. As a TCF/LEF protein belonging to the HMG box family, TCF7L1 is a major downstream effector of the Wnt signaling pathway and is an embryonic stem cell signature gene whose expression is upregulated in multiple aggressive cancer types, including breast cancer, glioblastoma, and bladder cancer [15,37]. Overexpression of miR-329-3p suppressed TCF7L1 expression, underscoring its role as a tumor suppressor in OS cells. This study presents the first evidence that targeted modulation of the miR-329-3p-TCF7L1-Wnt pathway axis in OS cells leads to a hindrance in the G1/S transition of the cell cycle, resulting in a slowed cell cycle progression and the initiation of apoptosis. Specifically, our data show that miR-329-3p inhibits OS cell proliferation, possibly by arresting the cell cycle in the G0/G1 phase. Western blotting indicated that the introduction of miR-329-3p inhibited Cyclin D1, which is downstream of the Wnt pathway. Cyclin D1 is a crucial cell cycle regulator that controls unchecked cell proliferation in the development of cancer [38]. Furthermore, the study of OS cytoskeletal dynamics revealed that the microfilaments in apoptotic OS cells were disintegrated, and that nuclear condensation and apoptotic bodies occurred. Additionally, miR-329-3p was inversely linked with TCF7L1 expression in sarcoma tissue and had a certain prognostic effect, according to the findings of an investigation of the TCGA database, which contained clinical data from 261 cases. Patients who had a low level of TCF7L1 expression had an overall survival rate that was significantly higher than patients who had a high level of TCF7L1 expression.

Studies of the Wnt pathway have shown that β-catenin is associated with TCF/LEF family transcription factors (TCF1, LEF1, TCF7L1, and TCF7L2), PYGO family co-activators (PYGO1 and PYGO2), and Legless family docking proteins (BCL9 and BCL9L) for the transcriptional activation of target genes of the Wnt-β-catenin signaling cascade, such as *MYC, CCND1, JAG1, DKK1, AXIN2* and *FGF20* [39]. Sustained activation of the Wnt/β-catenin pathway endows cancer cells the ability to proliferate continuously and self-renew, and it is linked to treatment resistance [40]. Several studies have demonstrated a direct interaction between TCF7L1 and β-catenin. One study used co-immunoprecipitation (co-IP) assays to show that TCF7L1 and β-catenin interact directly in mouse embryonic stem cells (mESCs) [41]. In addition, experiments using TCF7L1ΔN/ΔN knockin embryonic stem cells have further elucidated the role of the TCF7L1-β-catenin interaction in the regulation of gene expression [42]. Furthermore, crystallographic studies have provided molecular details of the interaction between TCF7L1 and β-catenin, revealing that the N-terminal domain of TCF7L1 binds to the armadillo repeat domain of β-catenin [43,44].

The experimental observation of miR-329-3p overexpression leading to an inhibitory effect on β-catenin expression raises intriguing questions about the underlying mechanisms. This phenomenon may be influenced by various factors, including post-transcriptional modifications, protein-protein interactions, or cross-talk with other signaling pathways. One plausible mechanism involves the ubiquitin-specific peptidase 22 (USP22)-mediated regulation of β-catenin expression. Indeed, Xin et al.’s investigation in hepatocellular carcinoma (HCC) presents compelling evidence of a regulatory interplay between miR-329-3p and USP22. Their comprehensive methodology, involving both bioinformatics analysis and experimental validation through HepG2 cell transfection, definitively established that miR-329-3p directly targets and suppresses USP22 expression. The credibility of this finding was further reinforced by a dual-luciferase reporter assay, which exhibited reduced luciferase activity in the presence of wild-type USP22 sequences [34]. Additionally, previous studies in colorectal cancer and gastric cancer have observed decreased total β-catenin levels upon inhibition of USP22 [45–47]. It is plausible to speculate that the miR-329-3p-USP22-Wnt/β-catenin axis may contribute to the observed downregulation of β-catenin expression in OS cell lines. Addressing the specificity of β-catenin inhibition, Fang et al. conducted experiments using the β-catenin specific inhibitor PRI-724 in a related study [48]. Their findings demonstrated a significant reduction in cell proliferation, migration, and invasion in human osteosarcoma cell lines 143B and SJSA-1, accompanied by a noticeable decrease in CyclinD1 expression. These results align with the outcomes of our miR-329-3p transfection experiments.

In addition to *in vitro* experiments, a tumor xenograft animal model of OS was employed to assess the *in vivo* antitumor effect of miR-329-3p. The study demonstrated that overexpression of miR-329-3p resulted in a substantial inhibition of OS xenograft tumor growth in nude mice. Immunohistochemical analyses conducted on tumor tissues further revealed a notable reduction in TCF7L1 and c-Myc expression within OS cells *in vivo*. The c-Myc encoded protein plays a pivotal role in intracellular signaling, orchestrating processes such as chromosomal DNA binding, cellular transformation, cell proliferation, differentiation, and apoptosis [49]. Its pivotal role in malignant transformation potentially underlies the observed tumor growth inhibition in the miR-329-3p treated groups. These findings collectively demonstrate that miR-329-3p exerts a substantial inhibitory impact on OS cell growth and promotes apoptosis by directly targeting TCF7L1, both *in vitro* and *in vivo*.

In conclusion, this study sheds light on the significant reduction in miR-329-3p expression within OS cells, an observation of paramount importance in governing the proliferation and cell cycle dynamics of OS cells. This regulatory function arises from the direct targeting of TCF7L1 by miR-329-3p, a phenomenon observed in both controlled *in vitro* settings and *in vivo* environments. Additionally, miR-329-3p suppresses the Wnt/β-catenin pathway, at least in part, to exert its anti-OS actions. This study marks the initial delineation of the connection between miR-329-3p and TCF7L1 in OS cells and provides a potential molecular underpinning for the genesis and progression of OS. The modulation of miR-329-3p expression could hold promise as a therapeutic approach for OS, warranting further exploration and clinical validation.

## Supplementary Materials

FIGURE S1 Genome-wide miRNA expression profiling conducted on a panel of cell lines including human mesenchymal stem cells (hMSCs) and five distinct osteosarcoma cell lines (MG-63, HOS, NY, SaOS, and Hu09).

FIGURE S2 Whole-genome mRNA profile illustrating the expression pattern of transcription factor 7-like 1 (TCF7L1) and its related genes in both human mesenchymal stem cells (hMSCs) and osteosarcoma cells. On the heat map depicting the relative expression, the color red indicates an upregulation, whereas the color blue indicates a downregulation.

FIGURE S3 Reciprocal correlation analysis between miR-329-3p and transcription factor 7-like 1 (TCF7L1) in The Cancer Genome Atlas (TCGA) data and its prognostic implications in sarcoma. Note the potential heterogeneity within sarcomas and osteosarcomas. Variations in gene expression profiles may arise due to the diverse molecular subtypes and genetic alterations present in these malignancies. (A) Co-expression investigation depicting the relationship between hsa-miR-329-3p and TCF7L1. miRNA-seq data-derived expression values were log2-scaled (log2(RPM + 0.01)). (B) Distribution pattern of miR-329-3p in sarcoma tissues, categorized based on high and low (50:50) TCF7L1 expression. (C) Assessment of the impact of miR-329-3p expression on overall patient survival in the context of sarcoma. (D) Evaluation of TCF7L1 expression's association with overall patient survival in sarcoma. The Wilcoxon test was utilized for intergroup statistical comparison. (****p < 0.0001).

FIGURE S4 Schematic representation of miR-329-3p. Depiction of the stem-loop structure of hsa-mir-329 precursor and the mature structure of miR-329-3p.

## Data Availability

The data and materials used in the present study are available from the corresponding authors upon reasonable request.

## Supplementary Materials

The supplementary material is available online at https://doi.org/10.32604/or.2023.044085.
