# Correction: MicroRNA-329-3p inhibits the Wnt/β-catenin pathway and proliferation of osteosarcoma cells by targeting transcription factor 7-like 1

**DOI:** 10.32604/or.2024.052652

**Published:** 2024-07-17

**Authors:** HUI SUN, MASANORI KAWANO, TATSUYA IWASAKI, ICHIRO ITONAGA, YUTA KUBOTA, HIROSHI TSUMURA, KAZUHIRO TANAKA

**Affiliations:** Department of Orthopaedic Surgery, Faculty of Medicine, Oita University, Oita, 879-5503, Japan

In the article ‘MicroRNA-329-3p inhibits the Wnt/β-catenin pathway and proliferation of osteosarcoma cells by targeting transcription factor 7-like 1’ (Oncology Research, 2024, Vol. 32, No. 3, pp. 463−476. doi: 10.32604/or.2023.044085), there was an error in the compilation of Fig. 8D. We have revised Fig. 8D to correct this error. A corrected version of Fig. 8 is provided. This correction does not change any results or conclusions of the article. We apologize for any inconvenience caused.

**Figure 8:**
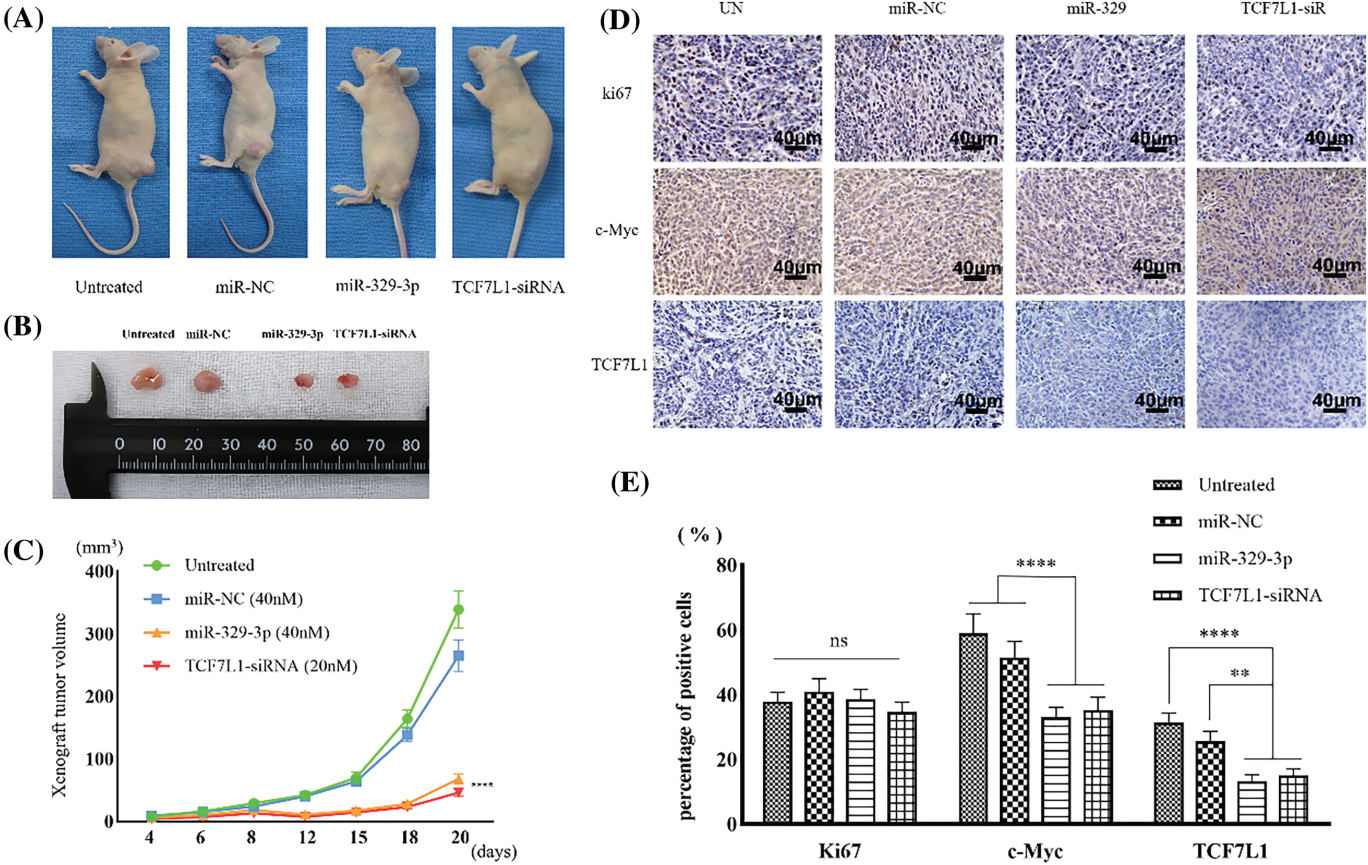
Xenograft tumors inhibited by miR-329-3p and TCF7L1-siRNA. (A) Photographs depicting the xenograft tumor. (B) Comparison of xenograft tumor size. (C) Following the inoculation of tumor cells, tumor volumes were measured at the indicated times. (D) TCF7L1, c-Myc, and ki67 expression changes in the xenograft tumor transfected with miR-329-3p and TCF7L1 siRNA. Magnification at origin: 200×; Scale bars = 40 μm. (E) Percentages of cells expressing ki67, c-Myc, and TCF7L1 in each group. One-way analysis of variance was employed to establish the significance of each group (n = 5). Tukey’s test was applied to correct for variance. Asterisks (*) denote the significance levels of the *p*-value, ns: *p* ≥ 0.05, **p < 0.01, ****p < 0.0001.

The authors would like to correct the figure as follows:

**Table:** 

Page. No.	Exact figure to be corrected	Correction
473	Fig. 8	Replace with new Fig. 8

The authors affirm that the scientific conclusions remain unaffected. This correction has been approved by the *Oncology Research* Editorial Office, and the original publication has been updated accordingly.

