# Supplementary figures and images for: MicroRNA-329-3p inhibits the Wnt/β-catenin pathway and proliferation of osteosarcoma cells by targeting transcription factor 7-like 1
Source: Oncol Res. 2024 Feb 6;32(3):463–76. doi: 10.32604/or.2023.044085 (PMC10874473; doi:10.32604/or.2023.044085)

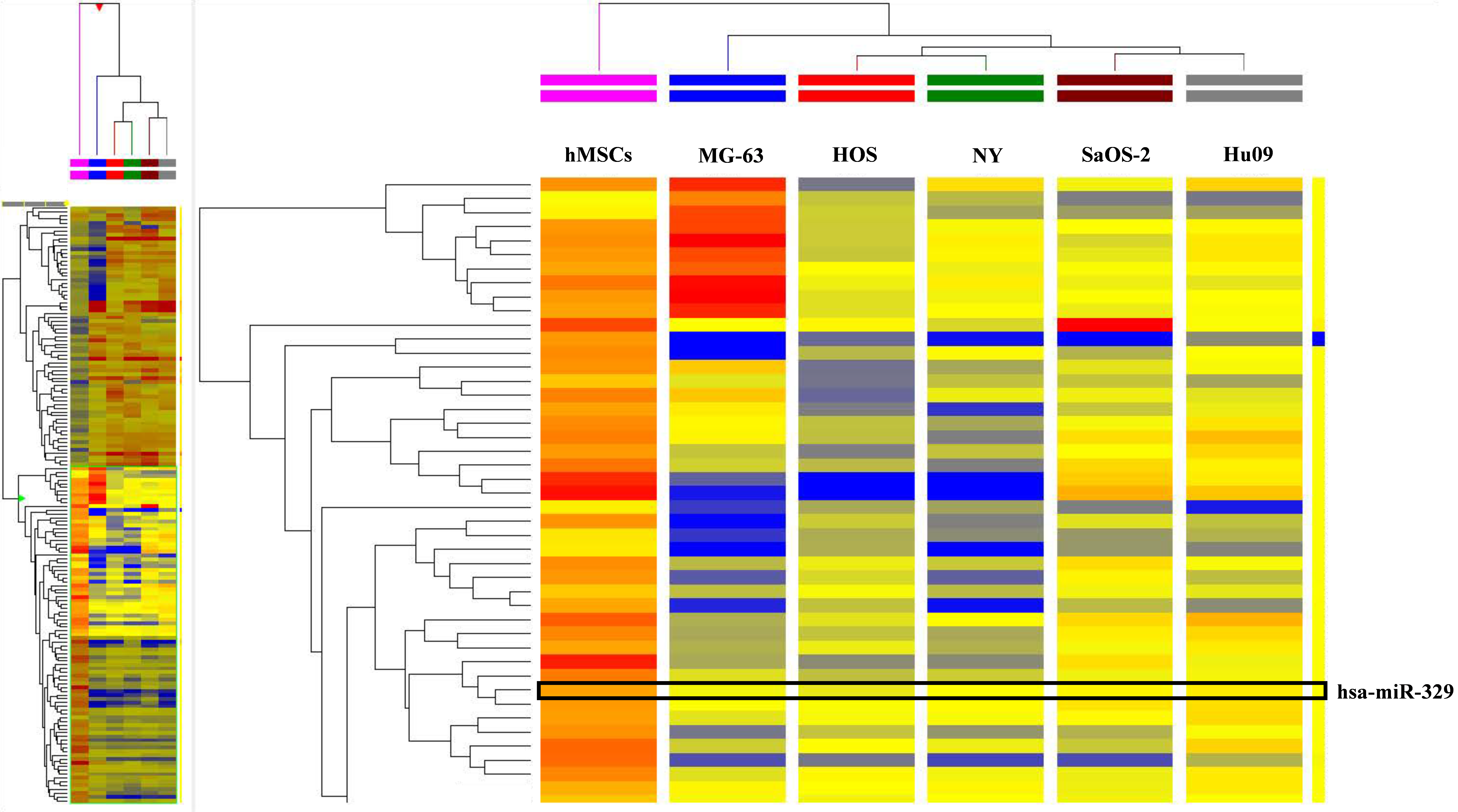

Supplement: FIGURE S1 [file OncolRes-32-44085-s001.tif]

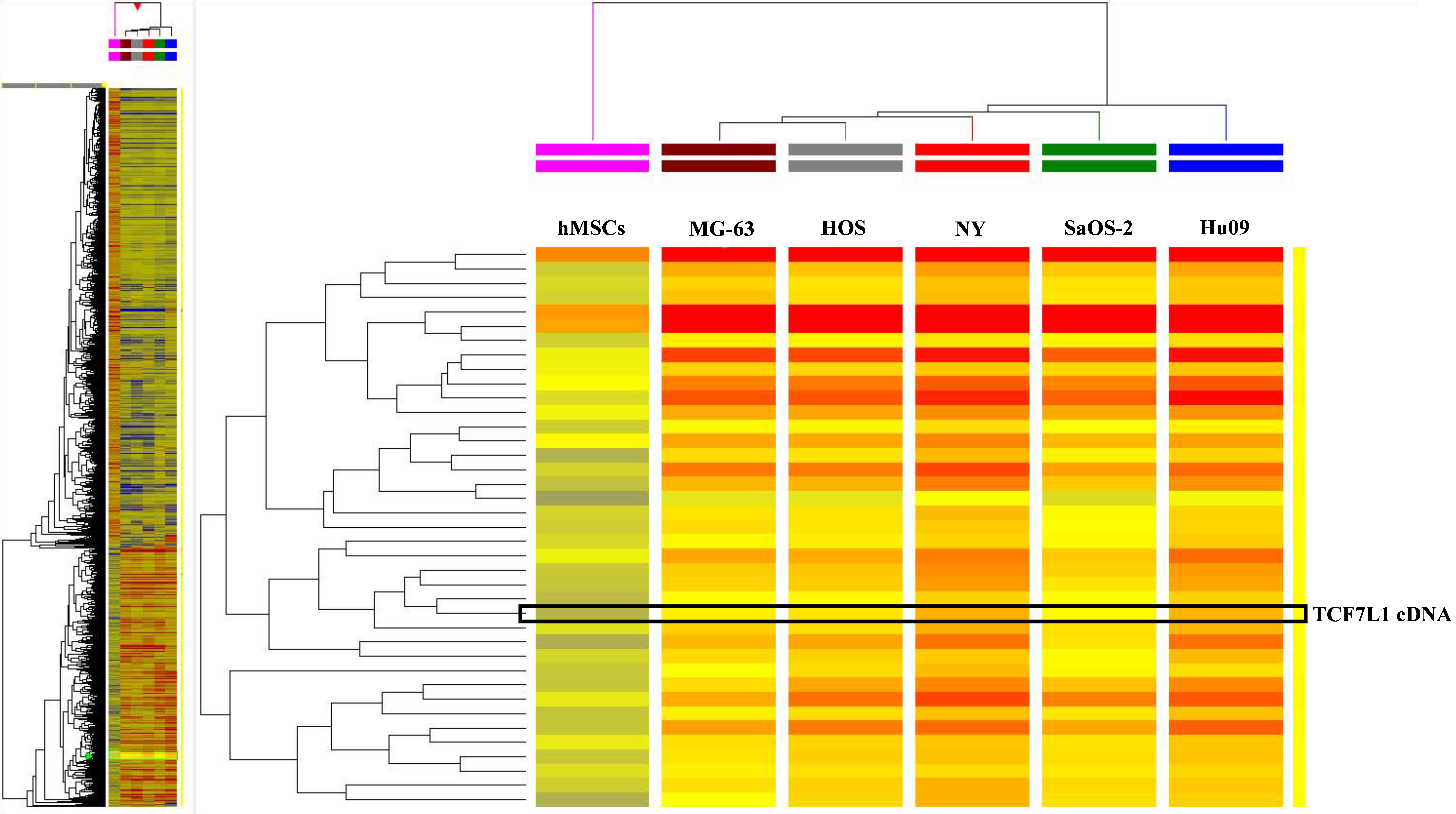

Supplement: FIGURE S2 [file OncolRes-32-44085-s002.tif]

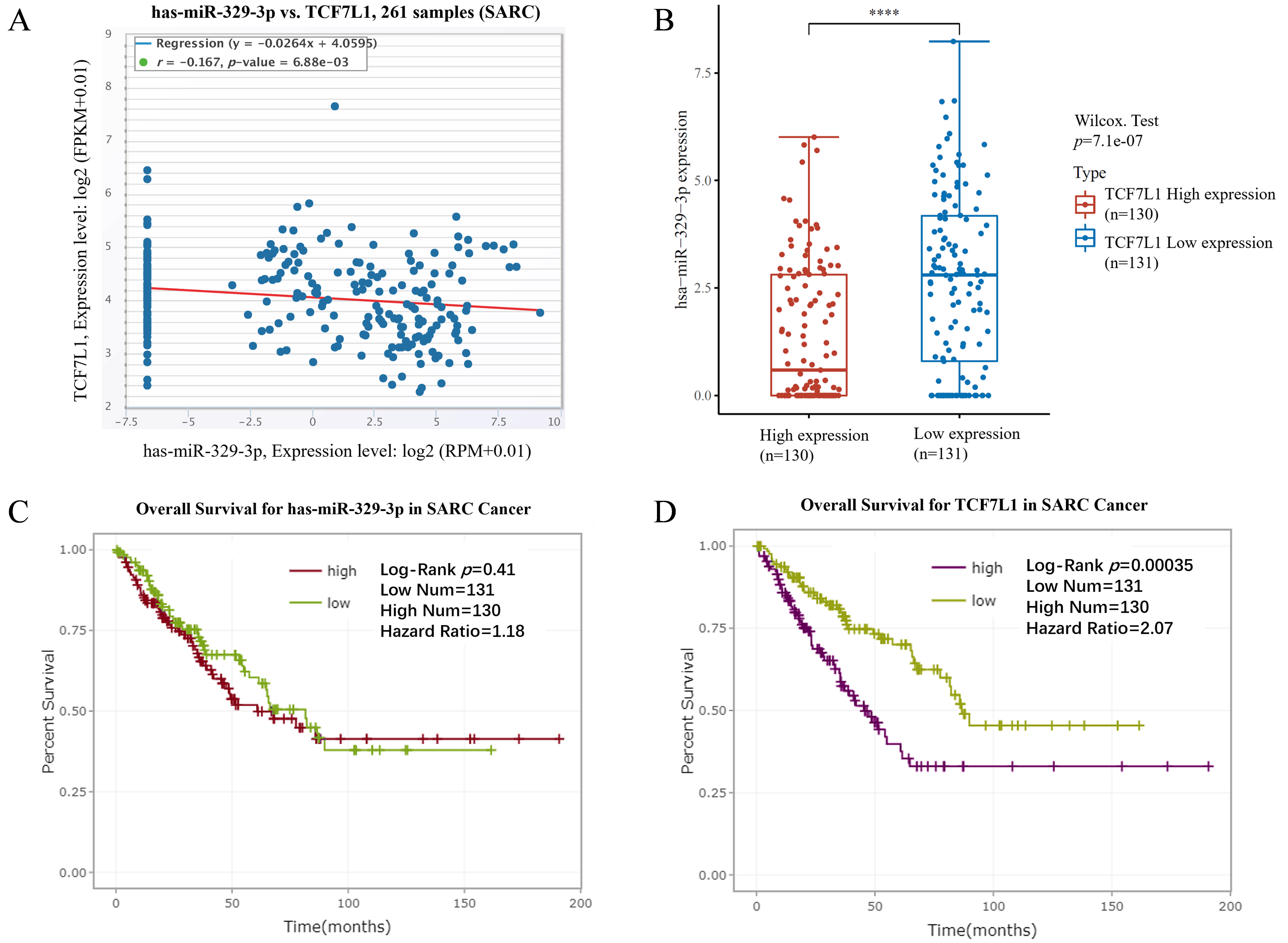

Supplement: FIGURE S3 [file OncolRes-32-44085-s003.tif]

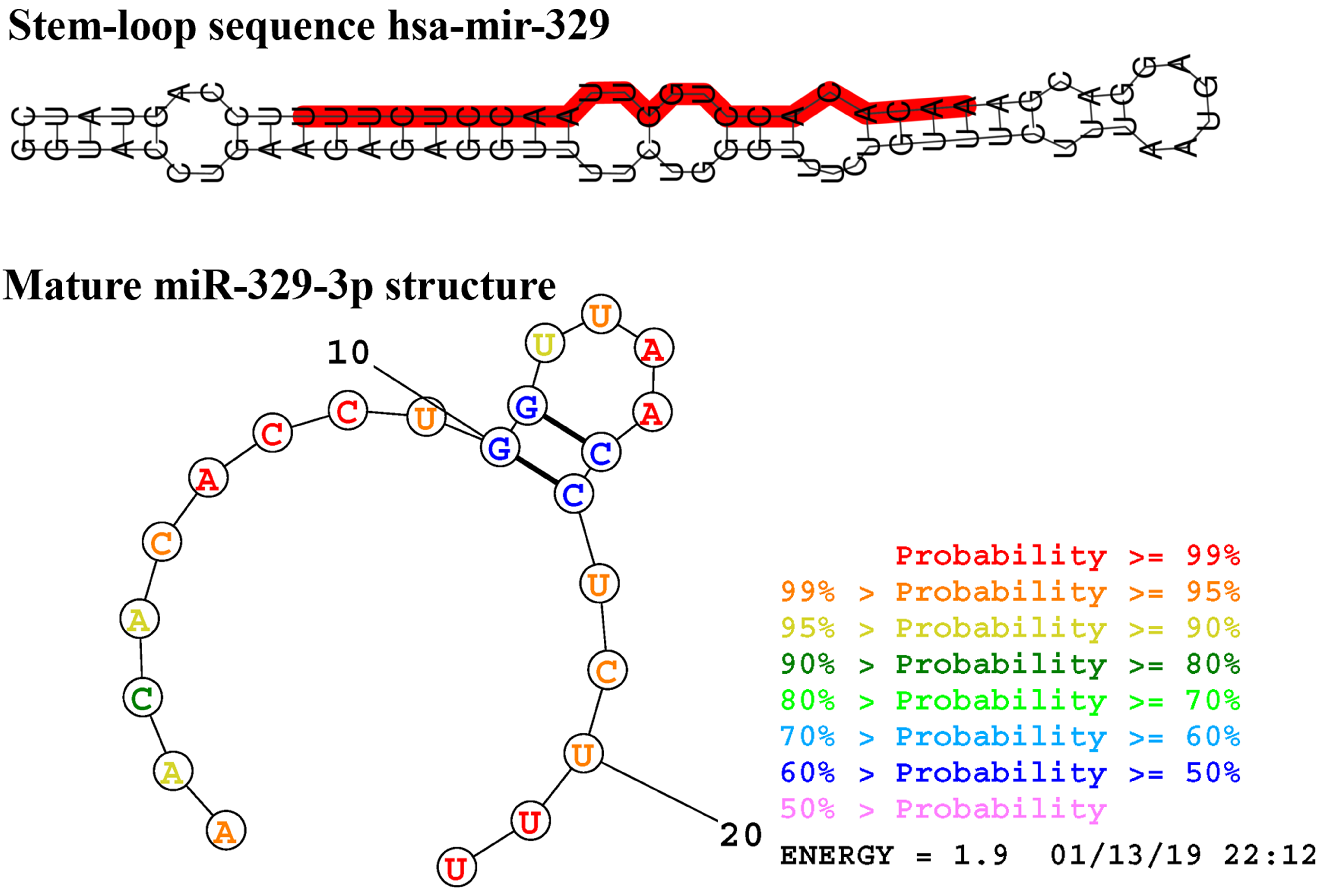

Supplement: FIGURE S4 [file OncolRes-32-44085-s004.tif]
